# Detection of human bocavirus-1 in both nasal and stool specimens from children under 5 years old with influenza-like illnesses or diarrhea in Gabon

**DOI:** 10.1186/s13104-018-3605-1

**Published:** 2018-07-20

**Authors:** Sonia Etenna Lekana-Douki, Sylvie Behillil, Vincent Enouf, Eric M. Leroy, Nicolas Berthet

**Affiliations:** 1 Centre International de Recherches Médicales de Franceville, BP 769, Franceville, Gabon; 2 Unité de Génétique Moléculaire des Virus à ARN, Centre National de Référence des Virus des Infections Respiratoires, Institut Pasteur, 25 rue du docteur Roux, Paris, France; 3 Centre National de Recherche Scientifique (CNRS) UMR3569, 25 rue du docteur Roux, Paris, France; 4 UMR (IRD 224/CNRS 5290/UM1-UM2), Institut de Recherche pour le Développement, Montpellier, France; 5 Unité Environnement et Risques Infectieux, Cellule d’Intervention Biologique d’Urgence, Institut Pasteur, 25 rue du docteur Roux, Paris, France

**Keywords:** HBoV-1, Influenza-like illness, Diarrhea, Children, Gabon

## Abstract

**Objective:**

Human bocavirus (HBoV) is a viral pathogen which causes respiratory tract diseases and acute gastroenteritis worldwide. This virus mainly affected children under 5 years old. There is little information on HBoV in Gabon. Two first studies was conducted to determine the prevalence of respiratory and enteric viruses in children under 5 years old who visited health centers for influenza-like illness (ILI) or diarrhea in Gabon from March 2010 to June 2011. However, HBoV was not included in the screening. The aim of this retrospective study was to evaluate the prevalence and the HBoV genotype in children under 5 years old with ILI or diarrhea in Gabon.

**Results:**

A total of 810 nasal swabs and 317 feces samples collected during the two first study were analyzed among which 32 (4.4%) and 7 (2.2%) were positive for HBoV respectively. While there were no significant differences in prevalence between age groups in children with ILI, all children with diarrhea were under 12 months of age. Moreover, 84.4 and 42.8% were diagnosed in co-infections with at least one other respiratory virus, or enteric viruses respectively. Finally, HBoV subtype 1 has been detected in both respiratory and gastrointestinal tracts with very low variability.

## Introduction

Pathogens causing respiratory tract infections and acute gastroenteritis have a significant impact on public health. Respiratory and gastrointestinal infections are the major causes of morbidity and mortality worldwide in children under the age of five [1, 2]. The human bocavirus (HBoV) was discovered in 2005 in children with respiratory infections [3]. HBoV is a single-stranded DNA virus, belonging to the family *Parvoviridae* and the subfamily *Parvovirinae*. HBoV-1 has been predominantly diagnosed in the respiratory tract where as the three other types, HBoV-2, HBoV-3 and HBoV-4, have been described as enteric viruses mainly excreted in stool [4]. Patients infected by the HBoV develop various clinical signs, such as rhinitis, pharyngitis, cough, dyspnea, wheezing, pneumonia, acute otitis media, fever, nausea, vomiting and diarrhea [5], and whose prevalence ranges from 5 to 10% of acute respiratory infections in children [6–8]. However HBoV can also be detected in an asymptomatic people [5].

In Central Africa, data on influenza-like illnesses (ILIs) are recent. HBoV has only been described in Cameroon in data showing a 10.6% prevalence [9].

In Gabon, several viruses were screening in two studies from March 2010 to June 2011. A total of 17 respiratory viruses [seasonal influenza virus A and B, pandemic influenza virus A(H1N1)pdm09, parainfluenza viruses (PIVs) types 1–4, respiratory syncytial virus (RSV), human metapneumovirus (hMPV), adenoviruses (AdV), rhinoviruses (HRV), enteroviruses (EV), coronaviruses HCoV-NL63, HCoV-HKU1, HCoV-OC43, HCoV-229E, and human parechovirus (HPeV)] were screened during this surveillance of ILIs among children under 5 years old [10]. Similarly, six enteric viruses [rotavirus A (RVA), adenovirus (AdV), norovirus I (NoVs-I) and II (NoVs-II), sapovirus (SaV) and human astrovirus (HAstV)] were screening during the same period in another study in children under 5 years old [11]. The most common viruses found were AdV (17.5%), PIVs (16.8%), EV (14.7%), RSV (13.5%), influenza viruses (11.9%) for ILI surveillance and RVA (27.1%), AdV (19.6%) and NoVs-II (13.9%) for diarrhea surveillance. Moreover, the prevalence of viral infection caused by at least one virus was 68.1 and 60.9% for ILI and diarrhea respectively. However, in those previous studies, HBoV was not included in the screening and few data are available on the circulation of this pathogen in Gabon. The main objective of this study were to evaluate the prevalence of HBoV in Gabon and to determine the genotype that circulate among children under 5 years old with ILI or diarrhea from March 2010 to June 2011.

## Main text

### Methods

#### Patients and samples

A retrospective study was conducted to evaluate the circulation of HBoV in children under 5 years old who visited health centers for ILI or diarrhea in Gabon. A total of 810 nasal swabs and 317 stool samples were collected in Libreville (the capital of Gabon), in Franceville (south-east), in Oyem (north) and in Koulamoutou (south). Demographical data such as the patient’s name, age, sex and clinical data including travel history during the month before onset were recorded. Nasal swabs “553C” (Copan, Diagnostic) placed in dry tubes and feces samples collected were stored at 4 °C until transportation to the *Centre International de Recherches Médicales de Franceville* (CIRMF), once a week for virological investigation. Seventeen respiratory viruses and six enteric viruses had previously been tested from their samples [10, 11].

#### Laboratory analysis

DNA was extracted on a BioRobot EZ1 workstation (Qiagen) using the EZ1 Virus Mini Kit version 2.0 (Qiagen) according to the manufacturer’s instructions [10]. Real-time polymerase chain reaction was performed in order to detect all types of HBoV using the forward primer 5′-CTGGGGCTCATATCATCA-3′, the reverse primer 5′-TCTCCCTCGTCTTCATCA-3′ and the probe 5′-AACACCCAATCAGCCACCT-3′ for the 93 bp virus nucleoprotein (NP) target. Each 20 µl of reaction mixture contained 5 µl of eluted DNA, 10 µl of Master Mix Fast (Applied Biosystems), 0.5 µM primers and 0.2 µM probe. A 7500 Fast Real Time PCR system was run for 2 min at 50 °C, for 20 s at 95 °C, followed by 50 cycles of 95 °C for 10 s and 60 °C for 30 s. For genotyping bocaviruses, the NP-1 gene fragment was amplified using a conventional PCR method (DNA Taq polymerase kit; Invitrogen) [3]. The generated amplicons (354 bp) were sequenced by the method of Sanger.

#### Phylogenetic and statistical analysis

A multiple sequence alignment of our bocavirus sequences with a selection of reference strains available in the GenBank database was performed using ClustalX (version 1.81). Phylogenetic relationships were reconstructed using the best-fitting ML model based on the Akaike information criterion, and General Time Reversible. The phylogenetic tree was built using the maximum-likelihood method with the PhyML algorithm [12–14] and drawn using FigTree v.1.4.0.

Statistical analysis was performed using Statview V5.0 software. Pearson’s Chi squared test and Fisher’s exact test were used to analyze the results. A two-tailed critical alpha value of 0.05 was used. A p-value below 0.05 was considered to indicate statistical significance.

### Results

#### Demographic data and prevalence of HBoV

Among the 810 patients with ILI, 416 were males (51.4%), 394 were females (48.6%) and the male:female (M:F) sex ratio was 1.05 (Table 1). The age ranged from 10 days to 4 years with median and mean ages of 1.33 years and 1.5 ± 1.0 years, respectively. Among these children, 341 (42.1%) were younger than 1 year old, 318 (39.3%) were 1–2 years old and 151 (18.6%) were 3–4 years old (Table 1).

**Table 1:** Demographic characteristics, prevalence of human bocavirus and co-infection

Characteristic	Human bocavirus (ILI)		Human bocavirus (diarrhea)	
	n1/N1 (%)	95% CI	n2/N2 (%)	95% CI
Sex				
Male	14/416 (3.4)	1.7–5.1	3/169 (1.8)	0.3–3.3
Female	18/394 (4.6)	2.5–6.7	4/148 (2.7)	0.9–4.5
Age group				
[0–12 m]	18/341 (5.3)	2.9–7.7	7/205 (3.4)	1.4–5.4
[13 m–2 y]	11/318 (3.5)	1.5–5.5	0/81 (0.0)	–
[3–4 y]	3/151 (2.0)	− 0.2 to 4.2	0/31 (0.0)	–
Towns				
Libreville	20/391 (5.1)	2.9–7.3	0/150 (0.0)	–
Franceville	7/159 (4.4)	1.2–7.6	1/56 (1.8)	0.3–3.3
Koulamoutou	1/98 (1.0)	− 1.0 to 3.0	1/33 (3.0)	1.1–4.9
Oyem	4/162 (2.5)	0.1–4.9	5/78 (6.4)	3.7–9.1
Total	32/810 (4.0)	2.7–5.3	7/317 (2.2)	0.6–3.8

Infection	n (%)		n (%)	
Single infection	5 (15.6)		4 (57)	
Dual infection	20 (62.5)		2 (28)	
Adenovirus	6 (22.3)		ND	
Enterovirus	4 (14.8)		ND	
RSV	3 (11.1)		ND	
Virus influenza B	3 (11.1)		ND	
Virus A(H1N1)pdm09	2 (7.4)		ND	
Rhinovirus	1 (3.7)		ND	
Coronavirus NL63	1 (3.7)		ND	
Rotavirus A	ND		1 (33.3)	
Sapovirus	ND		1 (33.3)	
Triple infection	7 (21.9)		1 (14.3)	
Enterovirus, rhinovirus	1 (3.7)		–	
Adenovirus, RSV	1 (3.7)		–	
Virus influenza B, HCoV-229E	1 (3.7)		–	
Adenovirus, enterovirus	1 (3.7)		–	
Enterovirus, HCoV-HKU1	1 (3.7)		–	
Virus A(H1N1)pdm09, HCoV-OC43	1 (3.7)		–	
PIV1, rhinovirus	1 (3.7)		–	
Adenovirus, norovirus 2	–		1 (33.3)	
Total coinfection	27 (84.4)		3 (43)	
Total	32 (100)		7 (100)	

*n1* number of bocavirus cases among patients with ILI, *N1* number of ILI cases, *n2* number of bocavirus cases among patients with diarrhea, *N2* number of diarrhea cases, *CI* confidence interval, *m* months, *y* years, *n* number of bocavirus, *ND* No data, *HCoV-229E* coronavirus 229E, *HCoV-HKU1* Coronavirus HKU1, *HCoV-OC43* Coronavirus OC43, *PIV1* parainfluenza virus 1

Among the 317 patients with diarrhea, 169 were males (53.3%), 148 were females (46.7%) and the male-to-female (M:F) sex ratio was 1.14. Patient ages ranged from 15 days to 4 years, with a median of 0.9 years and a mean of 1.1 ± 0.9 years. Among these cases, 205 (64.7%) were < 1 year old, 81 (25.5%) were 1–2 years old and 31 (9.8%) were 3–4 years old (Table 1).

The global prevalence of the HBoV was 4% (32/810) in the nasal specimens and 2.2% (7/317) in the stool samples (Table 1). The detection rate was similar in males and females in both types of infection, (X^2^ = 0.487, p = 0.49), (X^2^ = 0.032, p = 0.85). Moreover, while there were no significant differences between the age groups for detection of bocavirus in nasal samples (X^2^ = 3.3, p = 0.19), all children with diarrhea infected by HBoV were under 1 year old (Table 1).

#### Geographic and seasonal distribution of HBoV

**Fig. 1:**
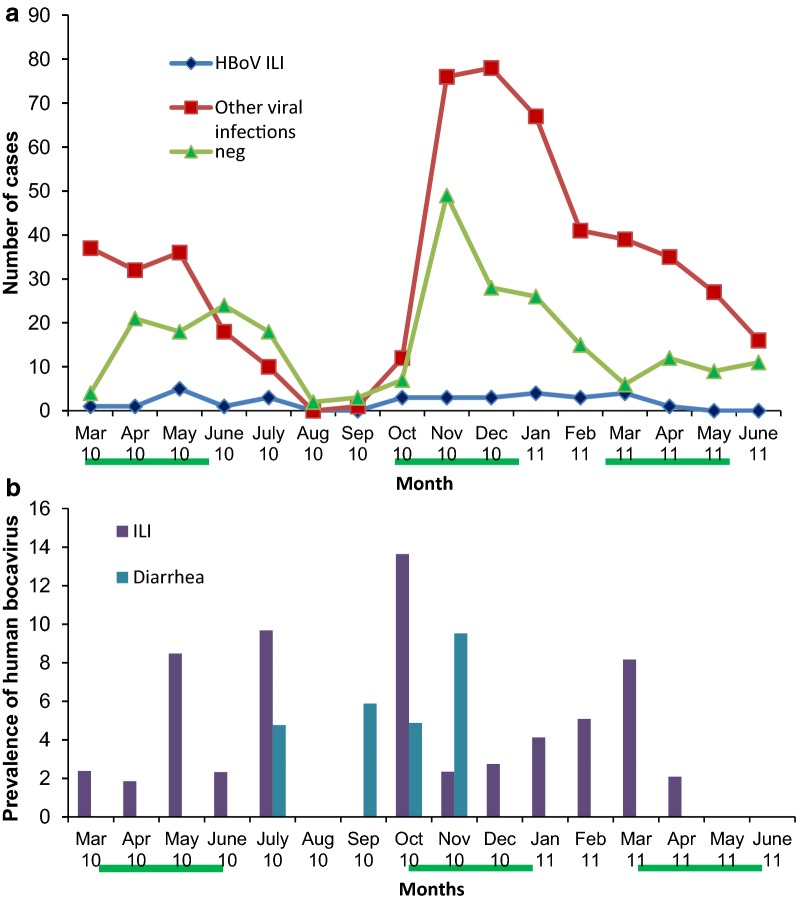
Human bocavirus in children under 5 years old in Gabon. **a** Number of cases among viral infections and influenza-like illnesses. **b** Prevalence of the human bocavirus during the different seasons among patients with ILI and diarrhea. The green line below the chart indicates the rainy seasons

Analysis of HBoV prevalence across the four towns showed no significant differences between Libreville (5.1%), Franceville (4.4%), Koulamoutou (1.0%) and Oyem (2.5%) for ILI (X^2^ = 4.6, p = 0.20) (Table 1). Among the 7 positive HBoV stool samples, bocavirus was detected most frequently in Oyem (5/7) (X^2^ = 9.9, p = 0.02), one case was detected in Franceville and one in Koulamoutou (Table 1). However, theses 7 cases were diagnosed during the big dry season and the short rainy season in 2010 (Fig. 1b). The number of ILI cases and the rate of virus-positive ILI increased during the rainy seasons (X^2^ = 7.0, p = 0.008) (Fig. 1a). However the rate of HBoV detection was low and did not vary significantly during the study (X^2^ = 0.002, p = 0.9) (Fig. 1b).

#### Coinfections

The bocavirus was detected in a single infection in 15.6% (5/32) of positive viral nasal samples; the remaining 84.4% (27/32) of the samples were detected in coinfection with another virus (Table 1). HBoV was detected predominantly in co-infections (X^2^ = 34.4, p < 0.0001). In this category, 62.5% (20/32) and 21.9% (7/32) were double and triple infections, respectively (Table 1). The most common co-infections were with AdV, followed by EV. Four (4/7) positive viral samples for HBoV showed a single infection, and three samples were co-infected with rotavirus A (n = 1), sapovirus (n = 1) and adenovirus/norovirus 2 (n = 1) (Table 1).

#### Phylogenetic analysis

**Fig. 2:**
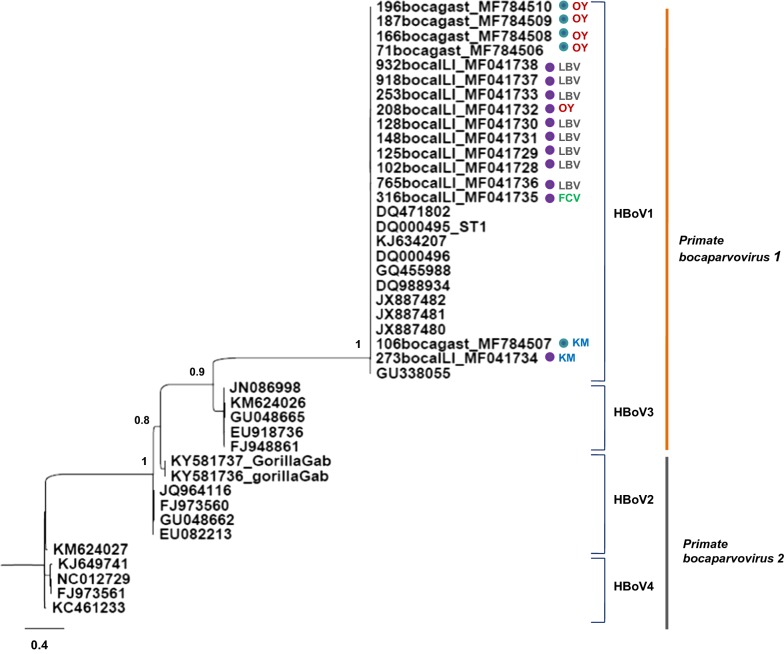
Phylogenetic tree based on 26 partial sequences of the nucleoprotein gene (NP1) belonging to the primate bocaparvovirus 1 and 2 species. The bootstrap values at node points were obtained using NNI (nearest neighbour interchange) and SPR (sub-tree pruning regrafting) branch swapping and 100 bootstrap replicates. The following letters indicates cities of the study. *OY* Oyem, *LBV* Libreville, *FCV* Franceville, *KM* Koulamoutou. The purple circles and the green circles indicate the strains detected among nasal and fecal specimens respectively

The phylogenetic analysis based on a partial sequence of the NP1 gene from 26 HBoV1–4 strains registered in the GenBank database showed that all 16 sequences obtained in our study (accession numbers MF041728-MF041738 and MF784506-MF784510, GenBank database) from nasal and stool specimens clustered with HBoV-1 (Fig. 2). Despite the small fragment sizes, two groups were discerned. In one group, 14 strains displayed 99% identity at the nucleotide level with the USA strain (Accession No. DQ471802) whereas the two remaining strains showed 100% identity at the nucleotide level with the reference strain from China (Accession No. JX887480) (Fig. 2). These latter sequences, that displayed one nucleotide difference with the others, were isolated from specimens collected in the same city Koulamoutou, one from a child with ILI and the other from a child with acute gastroenteritis (Fig. 2). The nucleotide substitution (T274A) on the NP-1 gene sequence generated a non-synonymous mutation, with an amino-acid substitution of serine with threonine.

### Discussion

In this study, HBoV prevalence was 4 and 2.2% in children under 5 years old with ILI and diarrhea, respectively, in Gabon. This low prevalence rate was similar to most previous studies, which have reported detection rates ranging from 5 to 10% [6–9, 15–18]. In our target population, influenza viruses, PIVs, RSV, HRV, AdV were generally diagnosed more frequently, as described in previous studies [19–21] and in Gabon [10]. Furthermore, worldwide, children under 5 years old with acute gastroenteritis are most frequently affected by rotavirus A (RVA) and norovirus (NoV) [22, 23]. Among the 317 stool samples of this study, the prevalence of enteric viruses was 21.7% for RVA, 19.6% for AdV, 9.1% for NoV-I, 13.9% for NoV-II, 9.5% for SaV and 6.3% for HastV [11].

Seroprevalence studies on HBoV confirms that the virus is acquired very early in life, before the age of two [7, 24]. This data corroborated our results showing that all children with diarrhea infected by HBoV were under 1 year old. However, we didn’t find any relationship between the virus that infected the patients and the severity of ILI (data not shown).

Our previous study showed that the viral infections of the respiratory tract are more frequent during the rainy season [10]. However, as observed in our study, a meta-analysis on HBoV infections failed to detect any seasonality on the African, Asian or American continents [9, 25]. The lack of evidence for seasonality maybe due either to very low prevalence or only sporadic cases of HBoV in several tropical countries [17, 26, 27].

In most studies, the rate of HBoV co-detection with at least one other virus ranges from 18 to 90% of respiratory samples [28]. Similarly, our study detected co-infection in 84.4% of HBoV infection cases. However, a study suggested that, this high rate of co-infection may challenge the role of HBoV as a causative pathogen, it may persist in the lymphatic tissue or become reactivated [29]. Viral loads tend to be higher in HBoV mono-infections than in co-infections [7, 28, 29]. We found that the threshold cycle values (Ct) were lower and the viral loads were therefore higher in monoinfection than in coinfection (data not shown). A global analysis of HBoV data shows co-detection in various frequencies with AdV, EV, HRV, RSV, influenza virus A/B and coronaviruses [28]. HBoV was found with frequently associated with rotavirus and norovirus [30, 31]. Similarly, our results showed coinfection with these previous viruses (Table 1).

The phylogenetic analysis of 16 strains showed that HBoV subtype 1 has been detected in both respiratory and gastrointestinal tracts, as described in China [32]. Studies mostly described HBoV-1 in respiratory specimens and HBoV-2, HBoV-3, HBoV-4 in stool specimens. However, other study reported that HBoV-1 was the most frequently genotype detected in stool specimens [33]. Finally our results are compatible with those of a Korean study showing all HBoV, detected in children with gastroenteritis and respiratory tract infection, were HBoV-1 genotype [34]. Our finding suggesting that the same HBoV-1 strain infected both respiratory and gastrointestinal tract supported the hypothesis that HBoV first causes respiratory tract infections and penetrates later to the gastrointestinal tract causing diarrhea [34]. Several studies showed that HBoV strains clustered into HBoV-1 with minor variations among them, sequences were highly conserved (99–100%) [34, 35]. This corroborated our results which show a very low variability of the HBoV-1 sequences.

In conclusion, this study provided the first data on the circulation of human HBoV in children under 5 years old in Gabon. The HBoV-1 genotype, was detected in both nasal and fecal specimens showing a low detection rate and similarly strains circulated in both respiratory and gastrointestinal infections. These data enhanced our knowledge on HBoV involved in respiratory infection and acute gastroenteritis and suggest that ILI and diarrheal surveillance should be continued to better understand the burden of these infections.

## Limitations

Considering the period of the study and the number of sites, the number of patients with ILI and diarrhea was probably underestimated.

## Abbreviations

HBoV: human bocavirus
ILI: influenza-like illness
PIVs: parainfluenza viruses
RSV: respiratory syncytial virus
hMPV: human metapneumovirus
AdV: adenovirus
HRV: rhinovirus
EV: enterovirus
HCoV: coronaviruses
HPeV: human parechovirus
RVA: rotavirus A
NoV-I: norovirus I
NoV-II: norovirus II
SaV: sapovirus
HAstV: human astrovirus
CIRMF: Centre International de Recherches Médicales de Franceville
NP: nucleoprotein
